# New insights into the Manila clam and PAMPs interaction based on RNA-seq analysis of clam through in vitro challenges with LPS, PGN, and poly(I:C)

**DOI:** 10.1186/s12864-020-06914-2

**Published:** 2020-08-01

**Authors:** Kunyin Jiang, Hongtao Nie, Dongdong Li, Xiwu Yan

**Affiliations:** 1 College of Fisheries and Life Science, Dalian Ocean University, Dalian, 116023 China; 2 Engineering Research Center of Shellfish Culture and Breeding in Liaoning Province, College of Fisheries and Life Science, Dalian Ocean University, Dalian, 116023 China

**Keywords:** Manila clam (*Ruditapes philippinarum*), Immune response, RNA-seq, DEGs, Pathogen-associated molecular patterns (PAMPs)

## Abstract

**Background:**

Manila clam (*Ruditapes philippinarum*) is a worldwide commercially important marine bivalve species. In recent years, however, microbial diseases caused high economic losses and have received increasing attention. To understand the molecular basis of the immune response to pathogen-associated molecular patterns (PAMPs) in *R. philippinarum*, transcriptome libraries of clam hepatopancreas were constructed at 24 h post-injection with Lipopolysaccharide (LPS), peptidoglycan (PGN), and polyinosinic-polycytidylic acid (poly(I:C)) and phosphate-buffered saline (PBS) control by using RNA sequencing technology (RNA-seq).

**Results:**

A total of 832, 839, and 188 differentially expressed genes (DEGs) were found in LPS, PGN, and poly(I:C) challenge group compared with PBS control, respectively. Several immune-related genes and pathways were activated in response to the different PAMPs, suggesting these genes and pathways might specifically participate in the immune response to pathogens. Besides, the analyses provided useful complementary data to compare different PAMPs challenges in vivo. Functional enrichment analysis of DEGs demonstrated that PAMPs responsive signal pathways were related to apoptosis, signal transduction, immune system, and signaling molecules and interaction. Several shared or specific DEGs response to different PAMPs were revealed in *R. philippinarum*, including pattern recognition receptors (PRRs), antimicrobial peptides (AMPs), interferon-induced proteins (IFI), and some other immune-related genes were found in the present work.

**Conclusions:**

This is the first study employing high throughput transcriptomic sequencing to provide valuable genomic resources and investigate Manila clam response to different PAMPs through in vivo challenges with LPS, PGN, and poly(I:C). The results obtained here provide new insights to understanding the immune characteristics of *R. philippinarum* response to different PAMPs. This information is critical to elucidate the molecular basis of *R. philippinarum* response to different pathogens invasion, which potentially can be used to develop effective control strategies for different pathogens.

## Background

Manila clam, *Ruditapes philippinarum*, is one of the most commercially important bivalves and reached over 4.2 million tons in 2017, which is widely distributed along the coast of China, Japan, and Korea [1]. *R. philippinarum* possesses many advantages as an aquaculture species, including wide salinity and temperature resistance, rapid growth, and pollution tolerance [2]. Nevertheless, *R. philippinarum* has been threatened with a huge challenge caused by pathogen invasion [3–5]. Pathogens can affect not only the development and survivorship of clams but also the quality and price of the product [6]. The majority of diseases in Manila clam are associated with Vibrio [7, 8] and Perkinsus [9, 10]. Diseases affecting *R. philippinarum* can result in mass mortality in aquaculture and cause large economic losses [11].

Although most bivalves lack a specific immune system, the innate response, which includes circulating hemocytes and multiple molecular effectors (PRRs, AMPs), appears to be an effective defense against external aggression [11]. Different receptors, regulators, and effectors, including pattern recognition receptors (PRRs), antimicrobial proteins (AMPs), and a variety of other molecules involved in agglutination, phagocytosis, and encapsulation, have been found in some Molluscs [12, 13]. In recent years, more and more research has been focused on the immune system of Molluscs [7, 14], and most of the research was focused on mussels, oyster, and scallop [15–17]. However, the available information on PRRs, AMPs, and immune-related signaling pathway of *R. philippinarum* is still limited.

In the past decades, the high-throughput RNA sequencing (RNA-seq) technique has been widely used to investigate molecular interactions between host and pathogen in Molluscs [6, 11]. Some efforts have been made to enrich the clam gene database and to gain an in-depth understanding of the potential immune mechanism of *R. philippinarum* [11, 18]. Recently, gene expression profiles of *R. philippinarum* hemocytes stimulated with *Perkinsus olseni* trophozoites, zoospores, and extracellular products under different experimental conditions were analyzed with RNA-seq on an Illumina platform [10]. The 454 pyrosequencing technology was used to obtain hemocytes transcriptome after in vitro immune-stimulated in the Manila clam, and a large number of immune-related genes were found that play important roles in the defense mechanisms of *R. philippinarum* [11]. Besides, transcriptional study in Manila clam in response to brown ring disease revealed that most changes in response to brown ring disease were tissue-specific, and a lot of candidate genes involved in microbe recognition and killing were identified [6]. Recently, the whole-genome of the Manila clam was assembled and annotated in our previous study [19], and the molecular basis of its adaptation to hypoxia, parasites and aerial exposure stress were analyzed [19–21]. However, the transcriptome analysis of response and defense against different pathogens or pathogen-associated molecular patterns (PAMPs) in Manila clam was still a largely unexplored landscape.

PAMPs, a class of conserved small molecular motif in microorganism, could be recognized by the PRRs of multicellular organisms and then activate innate immune response [22, 23]. Lipopolysaccharide (LPS), a well-characterized PAMP, is a component of the cell wall in Gram-negative bacteria [24]. It has been found that LPS could induce multiple innate immune responses in some Molluscs [25, 26]. Peptidoglycan (PGN), a component of the bacterial cell wall, is extracted from both Gram-positive and Gram-negative bacteria [23]. In *Crassostrea gigas*, PGN was recognized by peptidoglycan recognition proteins (*CgPGRPs*) and *CgTLR-6* [27, 28]. Polyinosinic-polycytidylic acid (poly(I:C)) is a kind of synthetic double-stranded RNA associated with viral infection [29]. It has been demonstrated that poly(I:C) could significantly up-regulate the expression of *CgIFNLP*, *CgIFNR-3*, and *CgCaspase*8–2 in *C. gigas* [30, 31]. In bivalves, pattern recognition molecules (PRMs) could recognize PAMPs and trigger the innate immune response [25, 27, 30, 31]. Hence, elucidating the immune response patterns and defense mechanisms of *R. philippinarum* against different PAMPs has important biological significance in the interpretation of the immune function of Manila clam.

In this study, we employed high throughput transcriptomic sequencing to investigate Manila clam response to different PAMPs through in vivo challenges with LPS, PGN, and poly(I:C). The hepatopancreas transcriptome of *R. philippinarum* after stimulated with three different PAMPs (LPS, PGN, and poly(I:C)) were analyzed to reveal the immune response of the Manila clam against LPS, PGN, and poly(I:C), and to elucidate the shared and specific immune-related genes in immune signaling pathways of Manila clam facing different PAMPs stress. This work sheds light on the molecular basis of Manila clam response to different PAMPs, and provides new insights into the immune signaling and pathogen defense responses of *R. philippinarum*.

## Results

### Genome-guided transcriptome assembly

A total of 533,660,306 raw reads were obtained, including 138,764,184 raw reads from the LPS treatment groups, 131,242,452 raw reads from the PGN treatment groups; 128,591,010 raw reads from the poly(I:C) treatment groups, and 135,062,660 raw reads from the PBS control groups corresponding to the constructed libraries. After low-quality reads (quality scores < 20), short reads (length < 60 bp), and ambiguous nucleotides were removed, a total of 266,830,153 clean reads were retained for further mapping and differential expression analysis. The clean reads were assembled into 35,919 unigenes with a mean length of 1681 bp, a minimum length of 122 bp, and a maximum length of 64,604 bp. Besides, 8266 novel genes were annotated in this study. Summarized trimming statistics and the number of sequenced reads per sample was shown in Additional file 1. The RNA sequencing data has been submitted to the NCBI SRA database (Accession number: PRJNA616201). Density distribution of expression level based on log10 (FPKM) in each library was exhibited in Additional file 2, which showed that LPS, PGN, and poly(I:C) groups were similar, while the PBS control group library was different with others three groups.

### Detection of differentially expressed genes

**Fig. 1:**
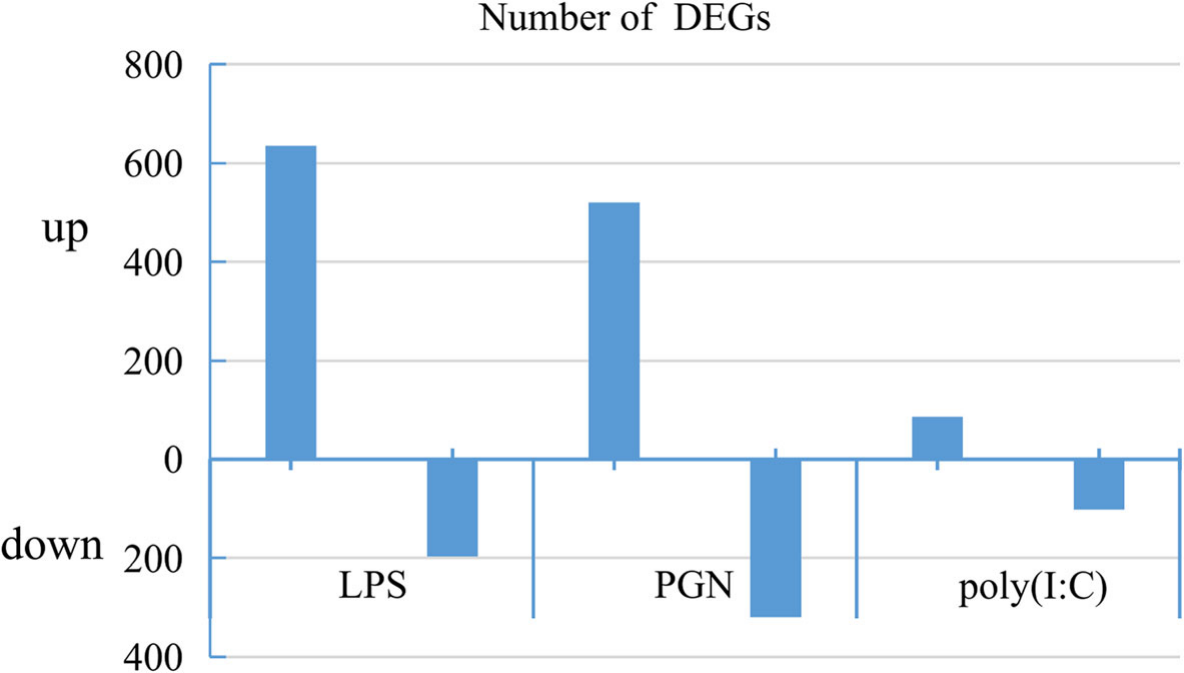
The number of up- and down-regulated DEGs in the hepatopancreas of *R. philippinarum* that was injected with LPS, PGN, poly(I:C). The DEGs number of LPS and PGN group was similar and was higher than the poly(I:C) group

A total of 1859 DEGs were identified from three PAMPs groups (LPS, PGN, and poly(I:C)) compared with the PBS group. The lowest number of DEGs (188) was found in the poly(I:C) group, while 832 and 839 DEGs were found in the LPS and PGN group, respectively. Of those DEGs, 635 up-regulated and 197 down-regulated in LPS group, 520 up-regulated and 319 down-regulated in PGN group and 86 up-regulated and 102 down-regulated in poly(I:C) group were identified compared with PBS control group, respectively (Fig. 1). The number of up-regulated genes were significantly higher than the down-regulated genes in LPS and PGN group.

### Genes activated by LPS, PGN, and poly(I:C)

**Fig. 2:**
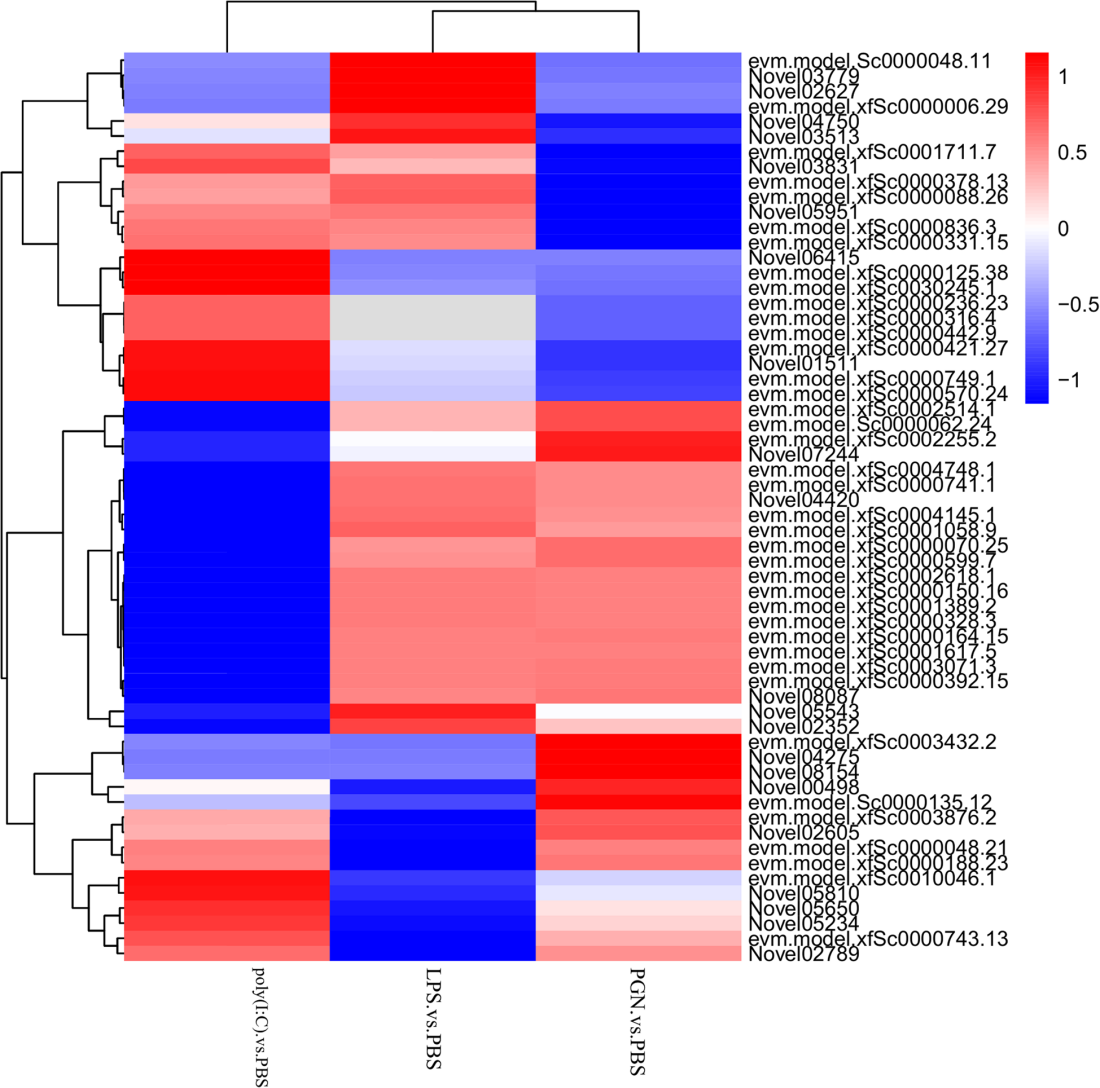
Heat map representing the differential expression (log2 fold change) of genes significantly induced in three independent experiments. Data show that the LPS group firstly clustered with the PGN group then with the poly(I:C) group

To uncover PAMPs responsive genes activated by LPS, PGN, or poly(I:C), the transcripts that passed the cut-off criteria (≥ 2 fold change, *P* < 0.05) were further analyzed and the known or putative function were shown in Table 1. A hierarchical clustering figure exhibited the global expression profiles of DEGs in each library (Fig. 2). It shows that the LPS group firstly clustered with PGN group then with the poly(I:C) group. The most up-regulated genes were defensin-B (*DEF*) and mucin-like protein (*MUCL*) in LPS group (26.8- and 25.5-fold, respectively), *DEF* and E3 ubiquitin-protein ligase (*RNF213*) in PGN group (26.3- and 25.7-fold, respectively) and cadherin EGF LAG seven-pass G-type receptor (*CELSR*) and Complement C1q tumor necrosis factor-related protein (*CTRP*) in poly(I:C) group (25.5- fold and 24.8-fold, respectively) (Table 1). Some pivotal PRMs directly involved in the innate immune system to reconigize PAMPs were significantly up- or down-regulated (≥ 5 fold) including peptidoglycan recognition protein (*PGRP*), toll-like receptor (*TLR*), ficolin (*FCN*), collectin (*CL*), complement c1q-like protein (*C1qL*), Complement component C3 (*C3*), calmodulin (*CALM*), cysteine-rich protein 2 (*CRIP2*), fibrinogen-like protein (*FREP*), serine protease inhibitor (*SPI*), interferon alpha-inducible protein (*IFI*), heat shock 70 kDa protein (*HSP70*), and etc. (Table 1).

**Table 1:** The shared and specific DEGs in Manila clam post injected with LPS, PGN and poly (I:C)

Abbreviations	Gene name	ID	Log2 Fold change		
			LPS vs PBS	PGN vs PBS	poly(I:C) vs PBS
DEGs shared by all three group					
*MUCL*	Mucin-like protein	evm.model.xfSc0001058.9	25.53	24.04	14.51
*IFI*	Interferon alpha-inducible protein 27	evm.model.xfSc0000070.25	10.58	11.38	3.46
*CTRP3*	Complement C1q tumor necrosis factor-related protein 3	evm.model.xfSc0000125.38	10.34	9.58	24.80
*Perlucin*	Perlucin	Novel03513	10.15	8.12	8.95
*CALM*	Calmodulin	evm.model.xfSc0002618.1	6.73	6.67	2.56
*LC*	Snaclec coagulation factor IX	evm.model.xfSc0030245.1	6.60	5.24	22.74
*DEF*	Defensin	Novel05650	4.90	8.49	11.01
*PGRP*	Peptidoglycan recognition protein	Novel01511	4.73	2.75	8.01
*ITGA*	Integrin alpha-4	Novel02605	3.64	6.31	5.72
*IFI*	Interferon alpha-inducible protein 27 like protein 1	evm.model.xfSc0000188.23	−23.45	−4.62	−5.44
*GBP1*	Interferon-induced guanylate-binding protein 1-like	Novel04750	−22.62	−23.78	− 23.09
*GTPA*	GTP-binding protein A	evm.model. Sc0000135.12	−9.97	−9.08	−9.72
*RNF213*	E3 ubiquitin-protein ligase RNF213	evm.model.xfSc0002255.2	−9.80	−5.56	−13.83
*NPC2*	Epididymal secretory protein E1	evm.model.xfSc0001403.6	−9.46	− 10.00	− 9.22
*HCK*	Tyrosine-protein kinase HCK	evm.model. Sc0000062.24	−8.90	−4.42	−22.62
*ITGB*	Integrin beta-like protein A	Novel03831	−7.15	−11.42	−5.63
*Ubr2*	E3 ubiquitin-protein ligase	evm.model.xfSc0002514.1	−6.90	−5.70	−10.69
*HAAF*	Hemagglutinin/amebocyte aggregation factor	Novel05234	−6.71	−2.32	−2.01
*LRIG1*	Leucine-rich repeats and immunoglobulin-like domains protein1	evm.model. Sc0000048.11	−6.16	−4.85	−4.57
*Ubr1*	E3 ubiquitin-protein ligase	Novel07244	−6.06	−5.34	−6.68
*DAPK1*	Death-associated protein kinase 1	evm.model.xfSc0000378.13	−4.94	−6.66	−5.17
*GBP1*	Interferon-induced guanylate-binding protein 1	Novel02352	−4.67	−5.99	−9.27
DEGs shared by LPS and PGN group					
*DEFB*	Defensin-B	evm.model.xfSc0000150.16	26.78	26.33	
*CDH23*	Cadherin-23	evm.model.xfSc0000164.15	10.73	10.79	
*HEXB*	Beta-hexosaminidase subunit beta	evm.model.xfSc0006767.1	8.71	9.95	
*NEK7*	Serine/threonine-protein kinase Nek7-like	Novel08087	8.22	8.64	
*CALM*	Calmodulin	evm.model.xfSc0000392.15	8.14	8.33	
*LR74A*	Leucine-rich repeat-containing protein 74A	evm.model.xfSc0000002.29	7.56	8.65	
*CRIP2*	Cysteine-rich protein 2	evm.model.xfSc0001224.15	5.90	6.92	
*GM2*	Ganglioside GM2 activator	evm.model.xfSc0000725.12	5.86	6.19	
*Nim1k*	Serine/threonine-protein kinase NIM1	evm.model.xfSc0000017.35	5.53	6.12	
*LR74A*	Leucine-rich repeat-containing protein 74A	evm.model.xfSc0000002.30	5.51	5.36	
*TEX14*	Serine/threonine-protein kinase TEX14	evm.model.xfSc0000164.11	5.20	5.64	
*CNR3*	Cell number regulator 3	evm.model.xfSc0001389.2	4.97	4.89	
*CSA1*	Cell surface antigen I/II	evm.model.xfSc0000741.1	4.80	4.25	
*TBA3*	Tubulin alpha-3 chain	evm.model.xfSc0000328.3	4.79	4.76	
*CATH*	Cathelicidin-B1	evm.model.xfSc0001753.3	4.65	4.09	
*SRCR*	Scavenger receptor cysteine-rich	evm.model.xfSc0004748.1	4.54	4.30	
*LSS*	Lysostaphin	evm.model.xfSc0000599.7	4.35	4.73	
*FCGR*	Low affinity immunoglobulin epsilon Fc receptor-like	Novel02789	3.78	7.03	
*DYH8*	Dynein heavy chain 8	evm.model. Sc0000012.11	3.68	5.54	
*FCGBP*	IgGFc-binding protein	evm.model.xfSc0000000.12	−9.374	−8.78	
*C1qL*	Complement C1q-like protein 4	evm.model.xfSc0010046.1	−7.39	−5.44	
*CTRP3*	Complement C1q tumor necrosis factor-related protein 3	evm.model.xfSc0001711.7	−2.30	−10.37	
*FA10*	Coagulation factor X	evm.model.xfSc0000836.3	−2.17	−8.98	
*CTL*	C-type lectin	evm.model.xfSc0000570.24	1.50	−2.74	
DEGs shared by LPS and poly(I:C) group					
*C1qL*	Complement C1q-like protein 4	evm.model.xfSc0000421.27	8.80		23.06
*CELSR1*	Cadherin EGF LAG seven-pass G-type receptor 1	evm.model.xfSc0000749.1	8.27		25.47
*THAP12*	52 kDa repressor of the inhibitor of the protein kinase-like	Novel02627	8.06		10.93
*HSP70A*	Heat shock 70 kDa protein 12A	evm.model.xfSc0002157.2	4.54		4.51
*C3*	Complement component C3	Novel05951	2.99		3.61
*TGFBI*	Transforming growth factor-beta-induced protein ig-h3	evm.model.xfSc0003432.2	−10.37		−10.09
*HSP70*	Heat shock 70 kDa protein	evm.model.xfSc0000048.21	−6.45		−7.55
*CL12*	Collectin-12	evm.model.xfSc0000743.13	−5.99		1.37
*ADGRE1*	Adhesion G protein-coupled receptor E1	evm.model.xfSc0003876.2	−5.77		−1.13
DEGs shared by PGN and poly(I:C) group					
*RNF213*	E3 ubiquitin-protein ligase RNF213	Novel00498		25.70	13.53
*MMR1*	Macrophage mannose receptor 1	evm.model.xfSc0000262.8		5.43	4.06
*PGRP*	Peptidoglycan recognition protein	evm.model.xfSc0000442.9		4.39	1.25
*TLR2*	Toll-like receptor 2	Novel05810		3.13	8.62
*GBP1*	Interferon-induced guanylate-binding protein 1-like	Novel03779		−25.11	−24.42
*Casp3*	Caspase-3	Novel00341		−6.27	−3.42
*FAS1*	Fasciclin-1-like isoform X2	Novel05543		−4.89	−9.62
*DD2*	Discoidin-2	Novel04420		−4.57	−25.07
*LTL*	L-type lectin	evm.model.xfSc0004145.1		−3.01	−10.70
Secific DEGs of LPS group					
*SPI*	Serine protease inhibitor Cvsi-2-like	Novel07619	12.47		
*FCN1*	Ficolin-1	evm.model.xfSc0000006.29	9.42		
*DYLC6*	Dynein light chain LC6	evm.model.xfSc0000411.8	9.33		
*HSP70B*	Heat shock 70 kDa protein 12B	evm.model.xfSc0003908.3	9.10		
*IMSP3*	Insoluble matrix shell protein 3	evm.model.xfSc0000186.20	8.98		
*IMSP3*	Insoluble matrix shell protein 3	evm.model. Sc0000176.1	5.71		
*IMSP1*	Insoluble matrix shell protein 1	evm.model.xfSc0000058.28	4.40		
*PAR14*	Poly [ADP-ribose] polymerase 14	evm.model.xfSc0003656.1	−24.49		
*PO*	Peroxidase-like protein	evm.model.xfSc0000759.9	−4.32		
Specific DEGs of PGN group					
*HSP70B*	Heat shock 70 kDa protein 12B	evm.model.xfSc0002250.4		22.30	
*HAAF*	Hemagglutinin/amebocyte aggregation factor	Novel08154		10.22	
*PYG*	Glycogen phosphorylase	evm.model.xfSc0001103.7		8.58	
*HSP70B*	Heat shock protein 70 B2	evm.model.xfSc0000005.7		8.87	
*DYH8*	Dynein heavy chain 8	evm.model. Sc0000012.12		5.87	
*TLR1*	Toll-like receptor 1	evm.model.xfSc0000331.15		−7.86	
*PAR14*	Poly [ADP-ribose] polymerase 14	evm.model.xfSc0001993.2		−6.92	
*CO8A*	Collagen alpha-1(VIII) chain	evm.model.xfSc0000095.25		−4.87	
*RNF213*	E3 ubiquitin-protein ligase RNF213	evm.model.xfSc0003464.1		−3.61	
Specific DEGs of poly(I:C) group					
*Siglec*	Sialic acid-binding lectin	Novel06415			24.00
*FCGR*	Low affinity immunoglobulin epsilon Fc receptor	evm.model.xfSc0000236.23			23.86
*IFI*	Interferon-induced protein 44-like	evm.model.xfSc0004748.1			11.07
*FREP*	Fibrinogen-like protein A	evm.model.xfSc0000316.4			10.07
*IFI*	Interferon-inducible GTPase 5-like	Novel03245			5.10
*IFI44*	Interferon-induced protein 44	evm.model.xfSc0003071.3			−25.60
*PAR12*	Poly [ADP-ribose] polymerase 12	evm.model.xfSc0000879.10			−24.32
*CAPRIN2*	Caprin-2	evm.model.xfSc0001617.5			−9.18
*LRR*	Leucine-rich repeat protein, putative	Novel03097			−5.82

### Comparison of transcriptome profiles elicited by different PAMPs

**Fig. 3:**
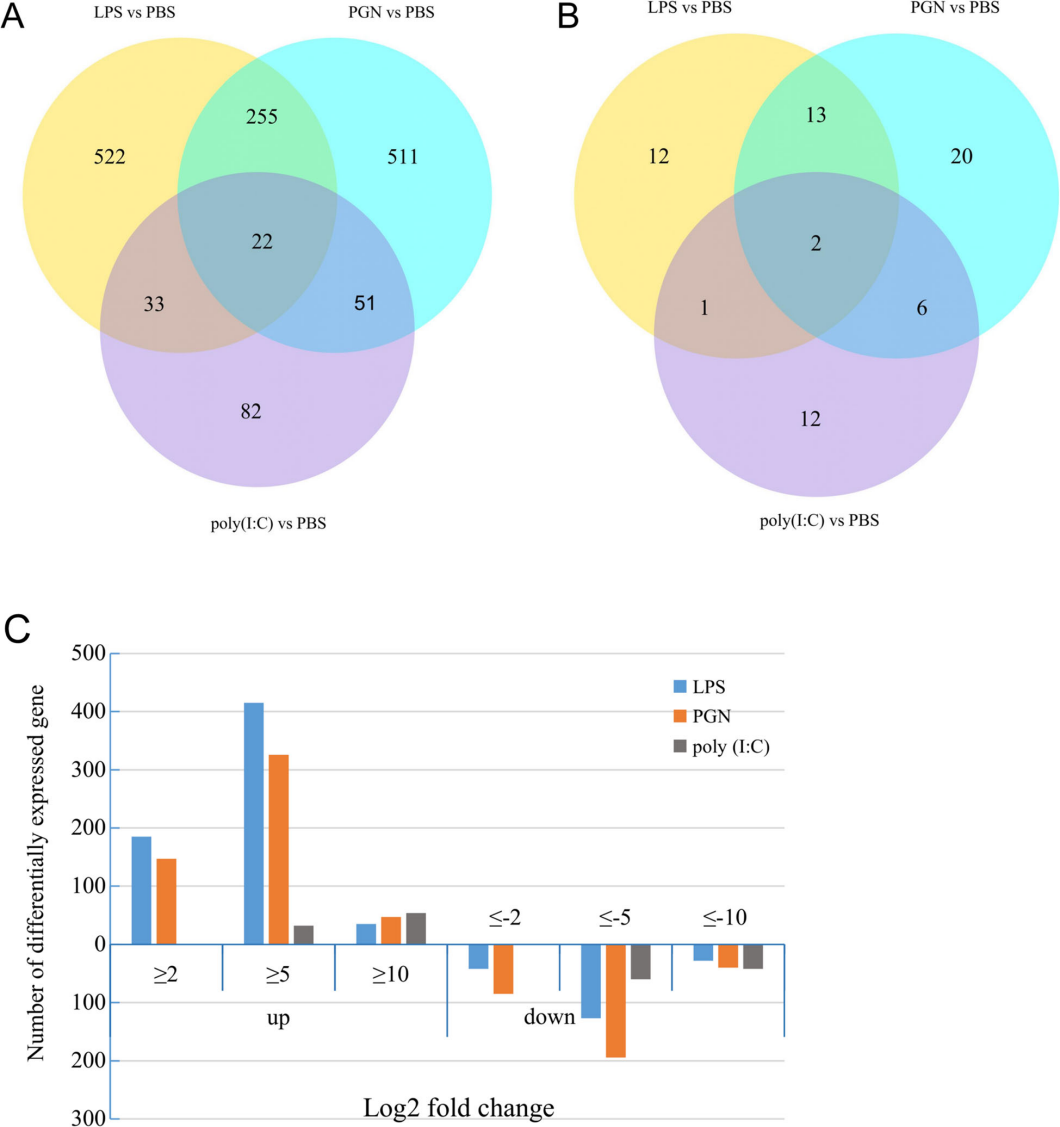
Venn diagram showing the overlap of pathways (**a**) and DEGs (**b**) regulated by the three PAMPs. The bar graph (**c**) showing the number of DEGs, grouping by the fold change. LPS and PGN induce a greater transcriptomic response than poly(I:C)

As showing in Fig. 3a and c, the DEGs in response to poly(I:C) were significantly less than that in response to LPS or PGN in *R. philippinarum*, while the number of DEGs in LPS and PGN groups were similar. A total of 255 genes were differentially expressed (≥ 2 fold change) in response to LPS challenge, which were also found in the DEGs of PGN challenge group, for example, Cathelicidin (*CATH*), scavenger receptor cysteine-rich (*SRCR*), IgGFc-binding protein (*FCGBP*), low affinity immunoglobulin epsilon Fc receptor (*FCGR*), Cell number regulator 3 (*CNR*). Besides, 33 DEGs in the LPS challenge group were also found in poly(I:C) challenge group, such as *CL*, *CELSR1*, transforming growth factor-beta-induced protein (*TGFBI*), and adhesion G protein-coupled receptor (*ADGRE1*). PGN challenge group and poly(I:C) challenge group shared 51 DEGs involved in PAMPs detection, including *TLR*, L-type lectin (*LTL*), macrophage mannose receptor *1* (*MMR1*), and discoidin-2 (*DD*2). Besides, there are 22 DEGs (fold change > 2) shared by all of the PAMPs group, such as *PGRP*, *Perlucin*, *C1qL*, *CTRP3*, *DEF*, *IFI*, and death-associated protein kinase (*DAPK*). (Fig. 3a, Table 1).

### GO and KEGG enrichment analysis of DEGs

**Fig. 4:**
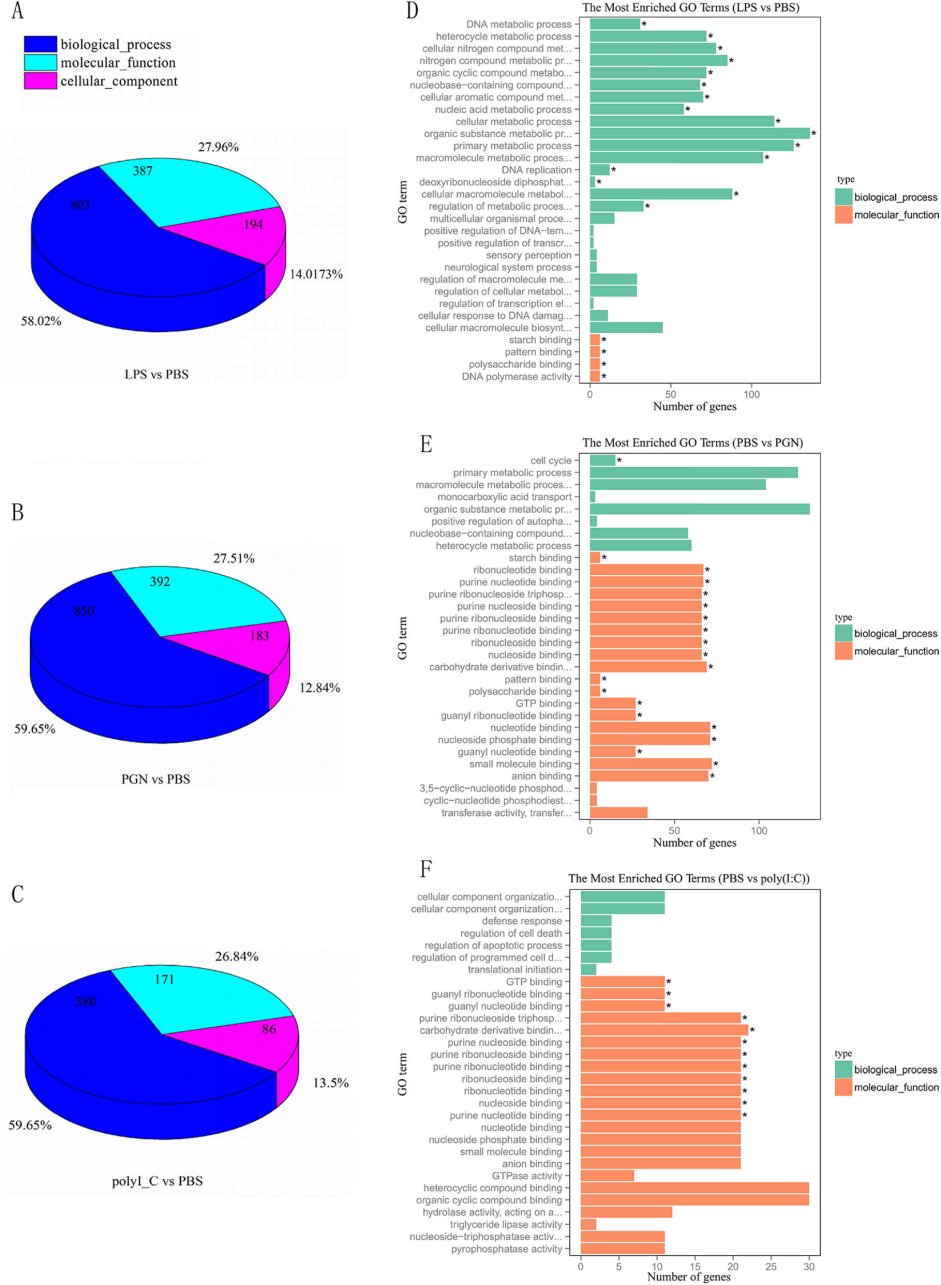
The proportion of biological process, molecular function, cell component (**a**, **b**, and **c**) and the distribution of enriched GO terms (**d**, **e**, and **f**) in the hepatopancreas transcriptome of *R. philippinarum* post LPS, PGN, poly(I:C) injection. The most enriched GO terms shared by those groups were primarily involved in immune response (GO:0006955), response to host immune response (GO:0052572), pattern binding (GO:0001871), positive regulation of GTPase activity (GO:0043547), and regulation of cell death (GO:0010941)

GO enrichment analysis was performed for the DEGs of each group. A total of 1384, 1452, and 637 terms were significantly enriched in LPS, PGN, and poly(I:C) group, respectively. The proportion of biological process, molecular function, cell component in those three group was similar (Fig. 4a-c). As showing in Fig. 4d-f, the top GO terms (the most enriched GO terms) shared by those group were primarily involved in immune response (GO:0006955), response to host immune response (GO:0052572), pattern binding (GO:0001871), positive regulation of GTPase activity (GO:0043547) and regulation of cell death (GO:0010941). In addition, some transcripts were clustered into the immune-related categories, such as response to stress (GO:0006950), response to bacterium (GO:0009617), response to wounding (GO:0009611), bacteriocin immunity (GO:0030153), wound healing (GO:0042060), and defense response to bacterium (GO:0042742). Those transcripts are likely to be involved in response to pathogens infection of *R. philippinarum*.

**Fig. 5:**
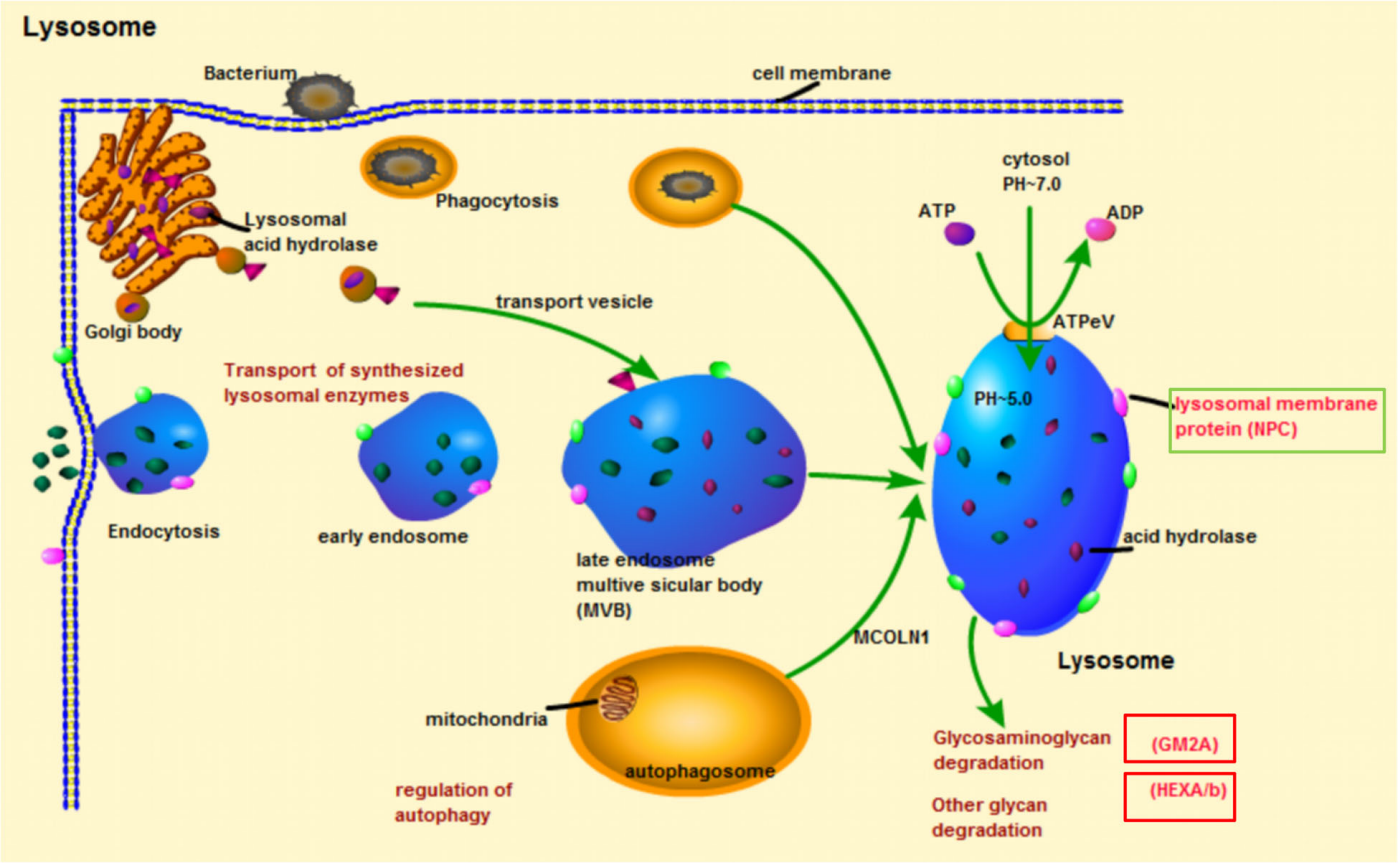
The Lysosome signaling pathway and DEGs in Lysosome pathway from KEGG enrichment analysis of *R. philippinarum* in LPS challenge group. The red colored genes were the DEGs in Lysosome pathway

**Fig. 6:**
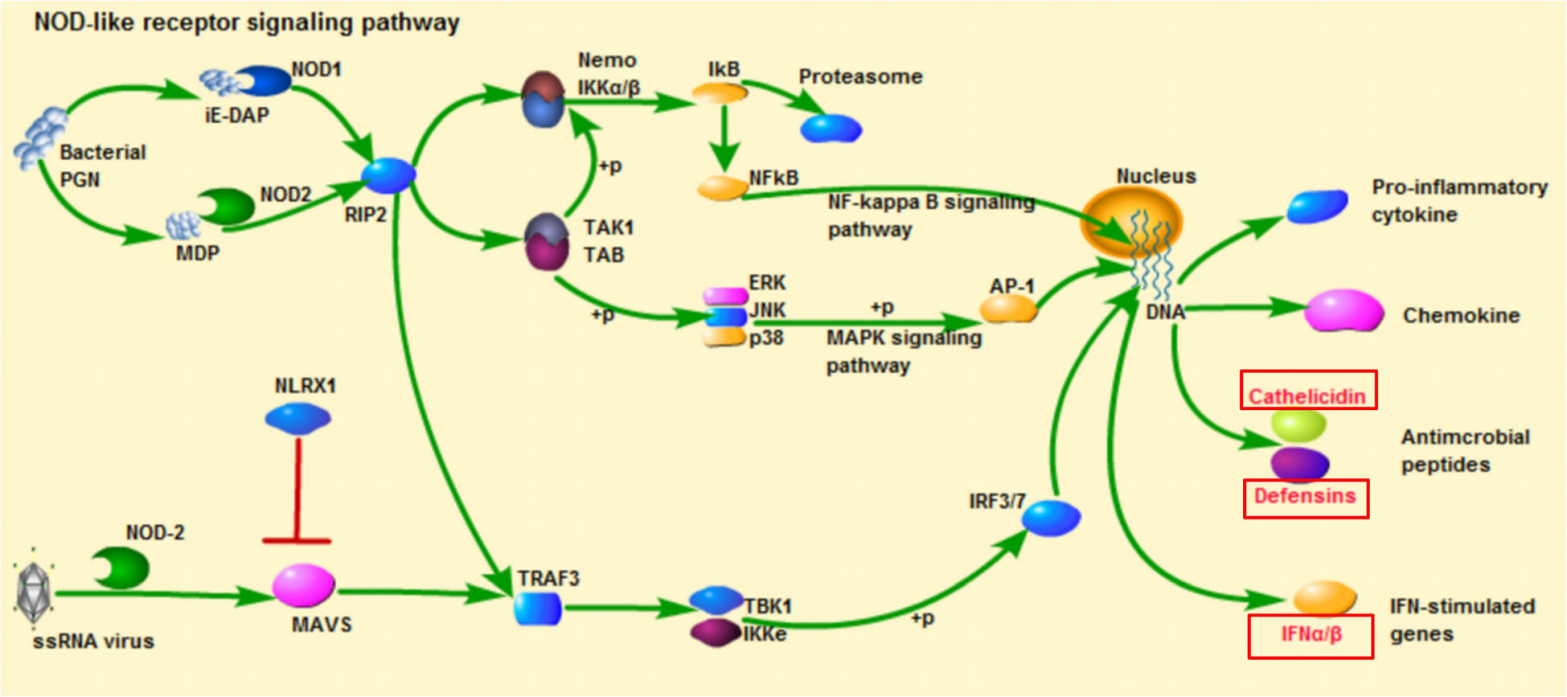
The NOD-like receptor signaling pathway and DEGs in NOD-like receptor signaling pathway from KEGG enrichment analysis of *R. philippinarum* in PGN challenge group. The red colored genes were the DEGs in NOD-like receptor signaling pathway

KEGG pathway enrichment analysis of DEGs was conducted to detect significantly altered pathways in each group. A total of 28, 41, and 21 pathways were significantly enriched in LPS, PGN, and poly(I:C) group, respectively (*P* < 0.05) (Fig. 3b). These pathways were primarily involved in apoptosis, signal transduction, immune system, and signaling molecules and interaction (Table 2). Furthermore, some enriched immune-related pathways were shared by multiple PAMPs groups, such as Platelet activation (map 04611) and Focal adhesion (map 04510) (shared by LPS, PGN, and poly(I:C) group), Phagosome (map 04145) (shared by LPS and poly(I:C) group), ECM-receptor interaction (map 04512) and Complement and coagulation cascades (map 04610) (shared by PGN poly(I:C) group), Cell cycle (map 04110), p53 signaling pathway (map 04115), cGMP-PKG signaling pathway (map 04022), and Calcium signaling pathway (map 04020), (shared by LPS and PGN group) (Table 2). Besides, some specific immune-related pathways enriched by DEGs also were identified, such as Lysosome (map 04142) (Fig. 5) and Regulation of actin cytoskeleton (map 04810) in LPS challenge group, NOD-like receptor signaling pathway (map 04621) (Fig. 6), Dopaminergic synapse (map 04728), Inflammatory mediator regulation of TRP channels (map 04750), and Melanogenesis (map 04916) in PGN challenge group; Cell adhesion molecules (CAMs) (map 04514) and B cell receptor signaling pathway (map 04662) in poly(I:C) challenge group (Additional file 3).

**Table 2:** The shared pathways enriched by DEGs among all 3 comparison groups (LPS vs PBS, PGN vs PBS and poly(I:C) vs PBS)

Pathway ID	Pathway	***P*** value	Class
Shared by LPS, PGN, and poly(I:C) group			
map 04611	Platelet activation	0.032959505	Organismal Systems; Immune system
map 04510	Focal adhesion	0.000108165	Cellular Processes
Shared by LPS and PGN group			
map 03030	DNA replication	1.47E-10	Genetic Information Processing; Replication and repair
map 04110	Cell cycle	2.54E-09	Cellular Processes; Cell growth and death
map 04113	Meiosis - yeast	1.67E-08	NO ENTRY FOUND.
map 04115	p53 signaling pathway	8.51E-07	Cellular Processes; Cell growth and death
map 03460	Fanconi anemia pathway	5.38E-06	Genetic Information Processing; Replication and repair
map 04971	Gastric acid secretion	8.67E-05	Organismal Systems; Digestive system
map 04921	Oxytocin signaling pathway	0.000627827	Organismal Systems; Endocrine system
map 04022	cGMP-PKG signaling pathway	0.000631031	Environmental Information Processing; Signal transduction
map 04270	Vascular smooth muscle contraction	0.002126258	Organismal Systems; Circulatory system
map 03410	Base excision repair	0.02148101	Genetic Information Processing; Replication and repair
map 04020	Calcium signaling pathway	0.032327041	Environmental Information Processing; Signal transduction
map 05166	HTLV-I infection	0.043004689	NO ENTRY FOUND.
map 00230	Purine metabolism	0.049496555	Metabolism; Nucleotide metabolism
Shared by LPS and poly(I:C) group			
map 04145	Phagosome	0.014509814	Cellular Processes; Transport and catabolism
Shared by PGN and poly(I:C) group			
map 05133	Pertussis	5.32E-05	Human Diseases; Infectious disease: bacterial
map 04974	Protein digestion and absorption	0.003941603	Organismal Systems; Digestive system
map 04512	ECM-receptor interaction	0.001614027	Environmental Information Processing; Signaling molecules and interaction
map 04610	Complement and coagulation cascades	0.000712509	Organismal Systems; Immune system
map 05130	Pathogenic *Escherichia coli* infection	0.013636368	Human Diseases; Infectious disease: bacterial
map 05322	Systemic lupus erythematosus	0.011539281	Human Diseases; Immune disease

### Validation of gene expression profiles using the quantitative real-time PCR

**Fig. 7:**
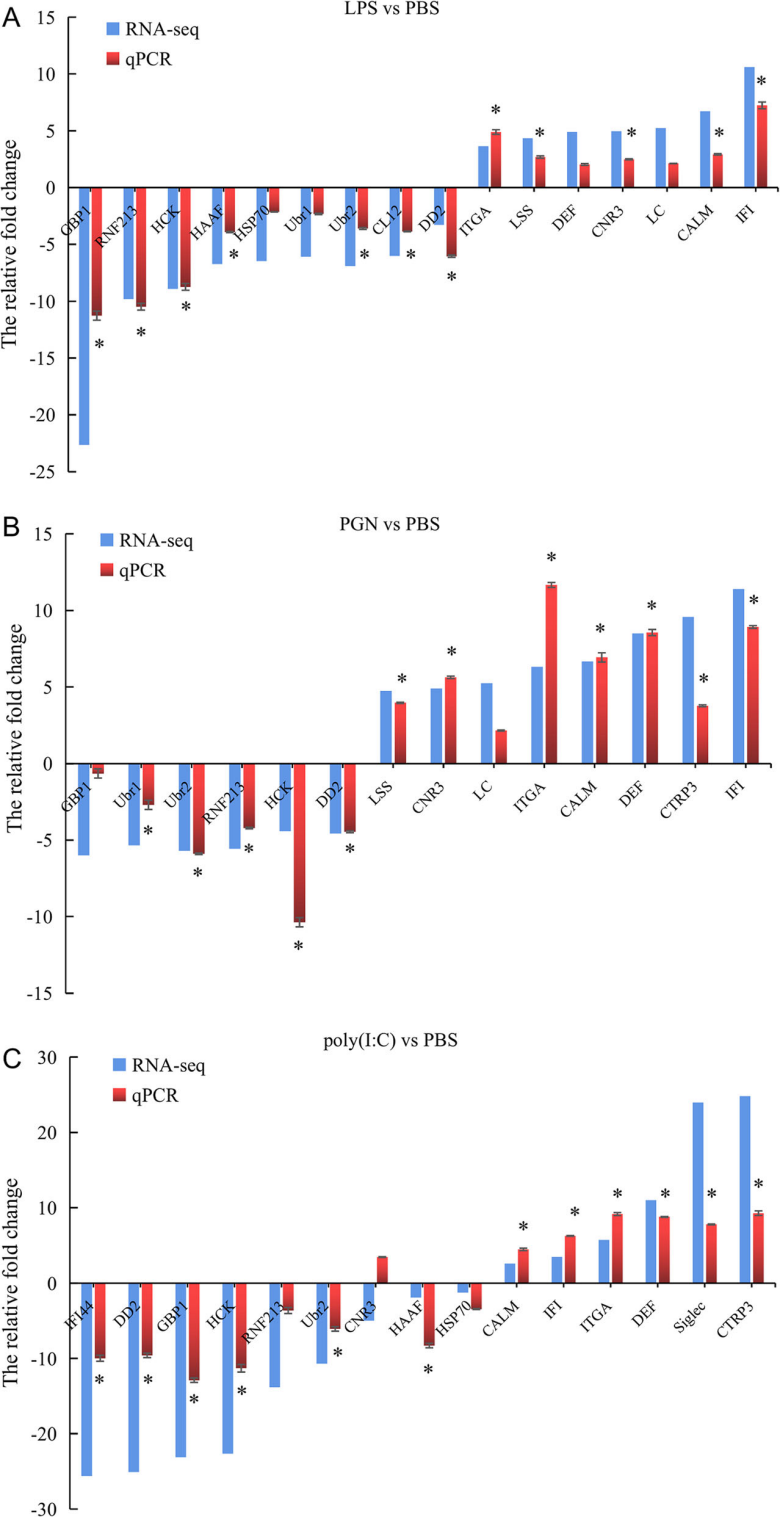
Validation of RNA-Seq results using qRT-PCR. The transcript expression levels of the selected genes were normalized to that of the *β-actin* gene

To validate the accuracy of RNA-seq results, we select both shared and specific immune-related genes that were differentially expressed in response to LPS, PGN, and poly(I:C) to perform the quantitative real-time PCR (qPCR) analysis. The specific primers of those genes were listed in Additional file 4. The fold change detected by qPCR was compared with that detected by RNA-Seq expression analysis (Fig. 7). As is shown in Fig. 7, nearly all of those DEGs shared the same trends in LPS, PGN, and poly(I:C) groups. In general, PAMPs responsive genes identified with quantitative real-time PCR experiments were consistent with the results of the Illumina sequencing analysis, indicating the accuracy of the RNA-seq expression analysis.

## Discussion

This study provides the first genome-based transcriptome analysis in the hepatopancreas of *R. philippinarum* under three different PAMPs challenge (LPS, PGN, and poly(I:C)). Due to the availability of *R. philippinarum* whole-genome sequence date [21], enable us could effectively extract reads that mapped to exons, avoid problems caused by intron-mapped reads [32], and found many novel genes which were not annotated in *R. philippinarum* genome. To better understand the innate immune system of *R. philippinarum* response to PAMPs challenge and to uncover the difference of Manila clam response to different PAMPs, the immune-related pathways, and genes that differentially expressed in different PAMPs challenge groups were analyzed. In the present study, several PRMs that are important in detecting PAMPs were identified, including the *FREP*, *C1qL*, *FCN*, *TLR*, *PGRP*, *Perlucin*, *MMR1*, C-type lectin (*CTL*), *CL*, *LTL*, *SRCR*, and sialic acid-binding lectin (*Siglec*) (Table 1), most of which could activate innate immune response by recognizing specific structures that are exposed to pathogens [22].

Lysozymes is a kind of alkaline enzyme that lyse bacteria by hydrolyzing the b-(1,4) linkage between N-acetylmuramic acid and N-acetylglucosamine of the peptidoglycan in bacterial cell walls [33]. The role of lysosomes in inflammatory response has been reported in Molluscan species [33, 34]. In bivalves, lysosomes were found within the granular hemocytes [35], and were released during degranulation of the granular haemocytes accompanies with phagocytosis [36]. The released enzymes then assist in the breakdown of foreign material [34]. It has been reported that a sub-lethal doses of the *Listonella anguillarum* (Gram-negative bacterium) tended to cause the destabilisation of the lysosomal membranes in *Ostrea edulis*, *C. gigas*, and *Pecten maximus* for a period of up to 48 h post-inoculation [35]. In the present study, the Lysosome pathway was conspicuously enriched between the LPS challenge group and the PBS control group (Fig. 5). In addition, five genes participating in the Lysosome pathways were significantly regulated in response to LPS challenge, indicating the Lysosome pathway plays a vital role in Gram-negative bacterium infection in *R. philippinarum*.

Intracellular PRMs, such as NOD-like receptor, could recognize virus-derived dsRNA and bacteria-derived peptidoglycan, and induce the inflammatory response [36]. More than 20 NOD-like receptors that play a key role in the recognition of intracellular ligands have been found in mammals [36]. Recently, it has been reported that Nod1 and Nod2 identify different sites of bacterial PGN and mediate innate immunity [37]. In Manila clam, the previous study reported that NOD-like receptor signaling pathway was enriched in the hepatopancreas transcriptome of *R. philippinarum* after challenged by *Vibrio anguillarum* [18]. In the current work, a total of 16 DEGs, such as *caspase*, *CATH*, *DEFs*, interferon-induced proteins (*IFI*), and Interferon-induced guanylate-binding proteins (*GBP*) in NOD-like receptor signaling pathway were highly regulated in response to PGN challenge in *R. philippinarum* (Fig. 6). Our finding indicated that NOD-like receptor signaling pathway might plays an important role in response to the Gram-positive bacterium in *R. philippinarum*.

Based upon the number of DEGs and pathways (Fig. 3a), *R. philippinarum* exhibited a stronger immune response to LPS and PGN challenge than to poly(I:C) challenge [38]. Interestingly, we found the transcriptomic responses of *R. philippinarum* to LPS and PGN shared more DEGs, including *C1qL*, *CTL*, *SRCR*, *CATH*, coagulation factor X (*FA10*), Leucine-rich repeat-containing protein (*LR74A*), etc. This result might be due to both LPS and PGN are derived from bacteria (Gram-negative and Gram-positive, respectively), whereas poly(I:C) is a synthetic analog of dsRNA associated with viral infection [29].

Some PAMPs, such as LPS and PGN, could activate TLRs and trigger the release of pro-inflammatory cytokines to induce immune response [39]. The present study demonstrates that PAMPs could significantly change the transcription level of immune-related genes involved in pathogen recognition (e.g. *FREP*, *C1qL*, *FCN*, *TLR*, *PGRP*, *Perlucin*, *MMR1*, *CTL*, *CL*, *SRCR*, *LTL*, *Siglec*) and killing (*C*3, *DEF*, *CATH*, *FCGBP*, *FCGR*), apoptosis regulation (*DAPK*, *CNR*, *caspase*), and stress response (*HSP*70). *FREP*, *C1qL*, *FCN*, *TLR*, *PGRP*, *Perlucin*, *MMR1*, *SRCR*, and Lectins (*CTL*, *CL*, *Siglec*, *LTL*) all belong to PRRs [12, 25], serve as dedicated sensors and/or exclusive effectors [40], and play a prominent role in activating intracellular signaling pathways and triggering the synthesis of antimicrobial effectors [41].

Among those PRRs, *C1qL* is described remarkably expanded in *R. philippinarum* [42]. It has been reported that some C1qL proteins are the first PRMs of the complement system from the evolutionary perspective [43]. Complement C1q-like proteins contain the C1q domain and were grouped as the C1qDC [43]. A total of 168 different transcripts of C1qDC was found in *Mytilus galloprovincialis*, most of which show differential expression following challenge with Gram-positive or -negative bacteria [44, 45], and the same expression pattern was also found in *Mytilus edulis* [46]. In this study, three *C1qL* genes were detected, and two *C1qL* genes showed up-regulated expression, while the other *C1qL* showed down-regulation. A similar result was found in *R. philippinarum* in response to brown ring disease [6] and other Molluscs [17, 45]. Therefore, It is conceivable that some *C1qL* transcripts are up-regulated while the others are down-regulated providing a tailored response to pathogens in *R. philippinarum* [6]*.* In addition, *C*3, *CTRP*, *FCGR*, and *FCGBP* also were detected highly regulated in PAMPs treatment groups. C3 functions as the key molecular of complement system to distinguish and eliminate pathogens, and induce inflammatory responses [47]. The membrane-attached *CTRP* is the initial prototype of C1q and acting as immune PRM activating the proto-complement [43, 44]. Besides, *FCN*, a derivative of the lectin pathway of complement activation [43], was found significantly up-regulated under LPS challenge in our study. To sum up, a rudimentary complement system with a group of expanded and diversified genes is suggested to exist in *R. philippinarum* and play a crucial role in the innate defense against pathogens [43].

*PGRPs* is considered to be a crucial immune molecule in Molluscs by detecting and eliminating invading bacteria [48–52]. The expression pattern of *PGRP* has been investigated in *Chlamys farreri* [49], *Solen grandis* [51], and *Hyriopsis cumingi* [52]. In this study, the up-regulated expression of *PGRP*s was not only detected in PGN challenged clams but also in LPS and poly(I:C) challenged clams (Table. 1). Similar results were found in *C. farreri* and *S. grandis* [49, 51]. In *C. farreri*, the *CfPGRP-S1* was a constitutive and inducible acute-phase protein that was involved in the immune response against both the Gram-positive bacteria and Gram-negative bacteria infection [49]. The expression of *SgPGRP-S2* was significantly up-regulated when *S. grandis* was stimulated by LPS, PGN, and b-1,3-glucan [51]. In Pacific oyster, a *CgPGRP-S1S* gene was found greatly contribute to efficient host defense systems, not only by direct interaction with bacteria, but also by triggering other defense pathways [50]. Therefore, we speculate *PGRP*s might not only serve as PRRs to recognize Gram-positive bacteria, but also participate in other defense pathways to respond to different pathogens invasion in *R. philippinarum.*

Immune effectors are usually induced by PRRs recognition and produced by epithelial cells from various organs, including AMPs, lysozymes, cytokines, antioxidant enzymes, and acute phase proteins [19]. In this study, two kind of AMPs (*DEF*, *CATH*) were annotated in DEGs in response to PAMPs. DEF is a large group of small antimicrobial peptides and involved in the host immune response against bacterial infection [53–57]. Various *DEF*s have been characterized in different bivalve species such as *Venerupis philippinarum* [53], *C. gigas* [54], *Argopecten irradians* [56], and *R. philippinarum* [55, 57]. In *C. gigas*, *Cg-def* gene exhibits high activities against Gram-positive bacteria but low activity against Gram-negative bacteria and fungi [54]. It has been reported *Rpdef* showed the highest activity against Gram-positive bacteria and played an important role in the elimination of invading bacterium through membrane-disruptive activity in *R. philippinarum* [55, 57]. In this study, two *DEF* (*defensin* and *defensin-B*) with six conserved cysteines were identified, which is consistent with previous reports of arthropod-like defensins in other Molluscs [54, 56, 58]. Both of the two *DEF* exhibited highly increasing expression in response to LPS and PGN challenge, especially the *defensin* gene increased 26.8- and 26.3-fold, respectively. The *defensin-B* showed a significantly up-regulated expression level in response to LPS, PGN, and poly(I:C). Our result indicates *DEF*s play vital roles in response to Gram-positive and -negative bacteria and virus invasion in *R. philippinarum*. *Cathelicidins* (*CATH*s) have broad antimicrobial activity against Gram-positive and Gram-negative bacteria in the insect [59]. However, little is known of the *CATH*s in bivalves, especially in *R. philippinarum*. In this study, the expression level of *Cathelicidin-B1* in *R. philippinarum* after LPS and PGN challenge was up-regulated (4.65- and 4.09-fold, respectively). We speculated that *CATH* is an essential molecule in *R. philippinarum* immunity system and may be utilized as executors for the incapacitation and elimination of Gram-positive and Gram-negative bacteria invasion.

Peroxidase-like protein (*PO*) belongs to the antioxidant enzymes [60]. Some reactive oxygen species (ROS) are highly harmful and toxic to organism and are significantly induced when attacked by invaders or stress [61, 62]. Organisms have formed an antioxidant defense system that removes ROS to protect cells from damage caused by abundant ROS [12]. In the present study, the *PO* gene was found significantly down-regulated (− 4.32 fold change) in the LPS group, indicating the efficiency of ROS removing was decreased and the immune system was affected in *R. philippinarum* after LPS challenge. Heat shock proteins (HSPs) are evolutionarily ancient and highly conserved intracellular molecular chaperones [19]. The primary role of HSP is to function as molecular chaperones to modulate stress response [12]. When organisms are stressed by environmental conditions, the expression level of *HSP* would significantly increase, enabling the organism could resist the damage caused by adverse environment to maintain homeostasis and cell survival [63, 64]. In this study, *HSP*s with up-regulated expression were found both in LPS, PGN, and poly(I:C) challenge group, indicating the activity of *HSP*s is closely linked to the innate immune system in *R. philippinarum* [65].

Another group of transcripts highly regulated in PAMPs challenged clams were interferon-induced proteins (*IFI*) and Interferon-induced guanylate-binding proteins (*GBP*). It has been reported that viral infection could trigger the interferon and interferon-induced genes highly up-regulated in salmonids and rainbow trout [66, 67]. In bivalves, *CgIFNLP* was found to increase significantly at 12 h (8.35-fold) and 24 h (4.95-fold) after poly(I:C) stimulation in *C. gigas* hemocytes [30]. GBP countered the antiviral effect by inhibition of its GTPase activity in Mammalian [68]. In this study, the expression of 5 *IFI* and 3 *GBP* genes was found highly regulated by poly(I:C) in *R. philippinarum* (*IFI* 3.46, 11.07, 5.10, –5.44, − 25.60, fold, and *GBP* -24.42, − 23.09, − 9.27 fold, respectively), while only 2 of *IFI* and 2 of *GBP* genes were regulated by LPS and PGN, and the expression level of those genes was similar. It is therefore plausible that some *IFI* were up-regulated, whereas others are switched down providing a tailored response to pathogens infection in *R. philippinarum*. Overall, these findings indicating *IFI* and *GBP* might play a crucial role in response to viral invasion in *R. philippinarum*.

## Conclusions

The transcriptome comparison of the different PAMPs challenged Manila clam has provided new useful data to understand the molecular basis of the immune response to pathogens. The genome-based transcriptome analysis revealed LPS and PGN are more potent PAMPs in activating the immune response in *R. philippinarum.* LPS challenge group shared more immune-related DEGs and immune response pathways with PGN challenge group than poly(I:C) stimulation. Besides, some significantly enriched specific pathways directly related to immune response were found, such as the Lysosome pathway in the LPS challenge group, NOD-like receptor signaling pathway in the PGN challenge group. Moreover, some PRRs (*FREP*, *C1qL*, *FCN*, *TLR*, *PGRP*, *Perlucin*, *MMR1*, *CTL*, *CL*, *SRCR*, *LTL*, *Siglec*), AMPs (*DEFs*, *CATH*), interferon-induced proteins (*IFI*, *GBP*), *HSP*s, and *PO* were identified, which play pivotal roles in identification and clearance of invading pathogens in *R. philippinarum.* Our finding will aid understanding of *R. philippinarum* immune system and defense response to different pathogens invasion and provide new insights to develop effective control strategies for different pathogens.

## Methods

### Manila clam and PAMPs challenge

The wild adult Manila clams used in this study were collected from Jinshitan, Dalian, Liaoning Province, China. The clams had an average shell length of 23.2 ± 1.0 mm, and an average weight of 5.4 ± 0.8 g. After being transported to the laboratory, the clams were cleaned to remove any fouling and were acclimated in aerated 20 L plastic tanks, containing water at 13.8 ± 0.6 °C, pH 8.1 ± 0.1 with a salinity of 30 ppt. The clams were fed with Spirulina powder once a day for 2 weeks and the water was exchanged fully once per day to discharge waste products. Clams in each group were fasted at least 2 days before injection to avoid food contamination.

Three PAMPs were used in this study, including LPS from the bacterium *Escherichia coli* 055:B5 (Solarbio, Beijing, China), PGN from *Staphylococcus aureus* (InvivoGen, USA), and synthetic dsRNA poly(I:C) (InvivoGen, USA). All three PAMPs were dissolved in 1 × PBS (phosphate-buffered saline, Solarbio, Beijing, China) at a concentration of 100 μg/mL (LPS and PGN) and 20 mg/mL (PGN) according to a previous study [69]. Clams were divided into three PAMP challenge groups including LPS group (LPS1, LPS2, LPS3), PGN group (PGN1, PGN2, PGN3), poly(I:C) group (poly(I:C)_1, poly(I:C)_2, poly(I:C)_3), and PBS control group (PBS1, PBS2, PBS3), respectively (each group *n =* 20). The clams from three PAMPs groups and PBS control group were injected into the sinusoid with approximately 50 μL of LPS (100 μg/mL), 50 μL of PGN (20 μg/mL), 50 μL of poly(I:C) (100 μg/mL), and 50 μL 1 × PBS, respectively. At 24 h post-injection, three clams in each group were randomly selected and the hepatopancreas was collected and immediately frozen in liquid nitrogen and stored at − 80 °C prior to use.

### RNA extraction and library construction for Illumina sequencing

Total RNA was extracted from 30 mg hepatopancreas of each individual (each group *n* = 3) using RNAprep pure Tissue Kit (TianGene, Beijing, China), according to the manufacturer’s protocol. The degradation and contamination of total RNA were monitored on 1% agarose gels. RNA purity and concentration were measured using the NanoPhotometer® spectrophotometer (IMPLEN, CA, USA) and Qubit® RNA Assay Kit in Qubit® 2.0 Fluorometer (Life Technologies, CA, USA), respectively.

A total amount of 3 μg RNA per sample was used as input material for the RNA sample preparations. Sequencing libraries were generated using NEBNext® UltraTM RNA Library Prep Kit for Illumina® (NEB, USA) following the manufacturer’s recommendations and index codes were added to attribute sequences to each sample [70]. Fragmentation was carried out using divalent cations under elevated temperature in NEBNext First-strand Synthesis Reaction Buffer (5X) [70, 71]. The first-strand cDNA was synthesized using random hexamer primer and M-MuLV Reverse Transcriptase (RNase H) [71]. Second strand cDNA synthesis was subsequently performed using DNA Polymerase I and RNase H. After adenylation of 3′ ends of DNA fragments, NEBNext Adaptor with hairpin loop structure was ligated to prepare for hybridization [71]. To select cDNA fragments of preferentially 250 ~ 300 bp in length, the library fragments were purified with AMPure XP system (Beckman Coulter, Beverly, USA) [72]. Then 3 μL USER enzyme (NEB, USA) was used with size-selected, adaptor-ligated cDNA at 37 °C for 15 min followed by 5 min at 95 °C before PCR. PCR was performed with Phusion High-Fidelity DNA polymerase, Universal PCR primers, and Index (X) Primer [72]. At last, PCR products were purified (AMPure XP system) and library quality was assessed on the Agilent Bioanalyzer 2100 system [72].

### Sequence filtering, mapping, and assembly

Raw reads of fastq format were firstly processed through in-house Perl scripts [73]. In this step, clean reads were obtained by removing reads containing adapter, reads containing ploy-N and low quality reads from raw data [73]. Clean reads were mapped to the reference genome of the *R. philippinarum* published in our previous study (https://www.ncbi.nlm.nih.gov/genome/?term=txid129788[orgn]) [21]. Index of the reference genome was built using Hisat2 v2.0.5 and paired-end clean reads were aligned to the reference genome using Hisat2 v2.0.5 [73]. We selected Hisat2 as the mapping tool for that Hisat2 can generate a database of splice junctions based on the gene model annotation file and thus a better mapping result than other non-splice mapping tools [74, 75]. Feature Counts v1.5.0-p3 was used to count the reads numbers mapped to each gene, and then FPKM of each gene was calculated based on the length of the gene and reads count mapped to this gene [76]. The *P* values were adjusted using the Benjamini & Hochberg method [77]. Next, the alignments are passed to StringTie (http://www.ccb. jhu.edu/ software/stringtie/) for transcript assembly. StringTie assembles the genes for each data set separately, estimating the expression levels of each gene and each isoform as it assembles them [78].

### Differential expression analysis

Differential expression analysis of two groups was performed using the DESeq2 R package [74]. DESeq2 provides statistical routines for determining differential expression in digital gene expression data using a model based on the negative binomial distribution [74]. The resulting *P*-values were adjusted using the Benjamini and Hochberg’s approach for controlling the false discovery rate [79]. Genes with an adjusted *P*-value < 0.05 found by DESeq2 were assigned as differentially expressed.

### GO and KEGG enrichment analysis of differentially expressed genes

Using transcripts from the reference genome [21], we annotated gene functions using the Gene Ontology (GO), and Kyoto Encyclopedia of Genes and Genomes (KEGG) database. GO enrichment analysis of differentially expressed genes (DEGs) was implemented by the cluster Profiler R package, in which gene length bias was corrected [80]. GO terms with corrected *P*-value less than 0.05 were considered significantly enriched by differential expressed genes [74]. KEGG is a database resource for understanding high-level functions and utilities of the biological system, such as the cell, the organism and the ecosystem, from molecular-level information, especially large-scale molecular data sets generated by genome sequencing and other high-throughput experimental technologies (http://www.genome.jp/kegg/) [74]. We used the cluster Profiler R package to test the statistical enrichment of differential expression genes in KEGG pathways [70].

### qPCR confirmation of Illumina sequencing data

To validate the Illumina sequencing data, twenty immune-related DEGs were chosen for quantitative real-time PCR (qPCR) analysis. The integrity and purity of RNA were determined by electrophoresis on a 1% agarose gel and a Nanodrop ND-2000 spectrophotometer (Thermo Electron Corp., Waltham, MA, USA), respectively. Total RNA was reverse-transcribed to cDNA with the PrimeScript RT reagent Kit (TaKaRa, Tokyo, Japan). The primers were designed with the Primer 5 software (Premier Biosoft International). The *β-actin* was selected as a reference gene for the qPCR analysis, due to its stably expressed characteristic [25, 26]. The qPCR was performed with TB Green Premix ExTaqII (TaKaRa, Tokyo, Japan). The reactions were carried out in a total volume of 20 μL containing 2 μL of diluted cDNA (50 μg/μL), 1 μL of each primer, 10 μL of TB Green PCR Master Mix and 6 μL of H_2_O, with the following cycling profile: 94 °C for 5 min, 40 cycles of 94 °C for 30 s, 60 °C for 30 s, and 72 °C for 30 s. Each sample was processed in triplicate in the Roche LightCycler 480 Real-Time PCR System (Roche Diagnostics Burgess Hill, UK). The 2^-ΔΔCT^ method [81] was used to analyze the expression level.

## Supplementary information

**Additional file 1. **Summary statistics of *R. philippinarum* transcriptome assembly. Q20, Q30: the percentage of bases with a Phred value of > 20 or 30.

**Additional file 2.** The density distribution of expression level of mapped clean read in LPS, PGN, poly(I:C), and PBS group.

**Additional file 3. **Kyoto Encyclopedia of Genes and Genomes (KEGG) assignment of unigenes in the transcriptome of *R. philippinarum* after LPS, PGN, poly(I:C) injection.

**Additional file 4.** Primers used for qPCR in this study.

## Data Availability

The transcriptome sequencing clean data have been submission to the SRA database in NCBI (https://www.ncbi.nlm.nih.gov/sra/?term=PRJNA616201) with accession number (PRJNA616201).

## Abbreviations

PAMPs: Pathogen-associated molecular patterns
LPS: Lipopolysaccharide
PGN: Peptidoglycan
poly(I:C): Polyinosinic-polycytidylic acid
PBS: Phosphate buffered saline
RNA-seq: RNA sequencing technology
DEGs: Differentially expressed genes
PRRs: Pattern recognition receptors
AMPs: Antimicrobial peptides
GO: Gene ontology
KEGG: Kyoto encyclopedia of genes and genomes
IFI: Interferon-induced proteins
PRMs: Pattern recognition molecules
DEF: Defensin
MUCL: Mucin-like protein
RNF213: E3 ubiquitin-protein ligase
CELSR: Cadherin EGF LAG seven-pass G-type receptor
CTRP: Complement C1q tumor necrosis factor-related protein
PGRP: Peptidoglycan recognition protein
TLR: Toll-like receptor
FCN: Ficolin
CL: Collectin
C1qL: Complement c1q-like protein
C3: Complement component C3
CALM: Calmodulin
CRIP2: Cysteine-rich protein
FREP: Fbrinogen-like protein
SPI: Serine protease inhibitor
IFI: Interferon alpha-inducible protein
HSP70: Heat shock 70 kDa protein
SRCR: Scavenger receptor cysteine-rich
FCGBP: IgGFc-binding protein
FCGR: Low affinity immunoglobulin epsilon Fc receptor
CNR: Cell number regulator 3
TGFBI: Transforming growth factor-beta-induced protein
ADGRE1: Adhesion G protein-coupled receptor
LTL: L-Type lectin
MMR1: Macrophage mannose receptor 1
DD2: Discoidin-2
DAPK: Death-associated protein kinase
Siglec: Sialic acid-binding lectin
NLR: NOD-like receptor
CTL: C-type lectin
FA10: Coagulation factor X
LR74A: Leucine-rich repeat-containing protein
PO: Peroxidase-like protein
ROS: Reactive oxygen species
HSPs: Heat shock proteins
