# Supplementary material for: New insights into the Manila clam and PAMPs interaction based on RNA-seq analysis of clam through in vitro challenges with LPS, PGN, and poly(I:C)
Source: BMC Genomics. 2020 Aug 1;21:531. doi: 10.1186/s12864-020-06914-2 (PMC7430831; doi:10.1186/s12864-020-06914-2)
Supplement: Supplementary file 1 — Additional file 1. Summary statistics of R. philippinarum transcriptome assembly. Q20, Q30: the percentage of bases with a Phred value of > 20 or 30. [file 12864_2020_6914_MOESM1_ESM.docx]

Additional file 1. Summary statistics of *R. philippinarum* transcriptome assembly. Q20, Q30: the percentage of bases with a Phred value of >20 or 30.

| Sample name | LPS_1 | LPS_3 | PBS_1 | PBS_2 | PGN_1 | PGN_2 | poly(I:C)_1 | poly(I:C)_3 |
| --- | --- | --- | --- | --- | --- | --- | --- | --- |
| Raw reads | 71,943,078 | 66,821,106 | 63,693,630 | 71,369,030 | 63,536,172 | 67,706,280 | 68,261,218 | 60,329,792 |
| Clean reads | 35,971,539 | 33,410,553 | 31,846,815 | 35,684,515 | 31,768,086 | 33,853,140 | 34,130,609 | 30,164,896 |
| Clean bases | 10.79G | 10.02G | 9.55G | 10.71G | 9.53G | 10.16G | 10.24G | 9.05G |
| Error rate (%) | 0.02 | 0.02 | 0.03 | 0.03 | 0.03 | 0.02 | 0.03 | 0.03 |
| GC content (%) | 36.33 | 36.35 | 39.04 | 35.83 | 37.75 | 37.78 | 34.85 | 38.03 |
| Total reads | 71,943,078 | 66,821,106 | 63,693,630 | 71,369,030 | 63,536,172 | 67,706,280 | 68,261,218 | 60,329,792 |
| Total mapped | 34,889,781 (48.50%) | 31,369,926 (46.95%) | 36,140,231 (56.74%) | 33,467,364 (46.89%) | 35,222,166 (55.44%) | 36,603,251 (54.06%) | 32,345,998 (47.39%) | 31,544,558 (52.29%) |
| Q20 (%) | 98.18 | 98.19 | 98.02 | 97.92 | 98.10 | 98.26 | 97.83 | 97.83 |
| Q30 (%) | 94.29 | 94.39 | 94.00 | 93.80 | 94.11 | 94.50 | 93.38 | 94.10 |
