# Supplementary figures and images for: New insights into the Manila clam and PAMPs interaction based on RNA-seq analysis of clam through in vitro challenges with LPS, PGN, and poly(I:C)
Source: BMC Genomics. 2020 Aug 1;21:531. doi: 10.1186/s12864-020-06914-2 (PMC7430831; doi:10.1186/s12864-020-06914-2)

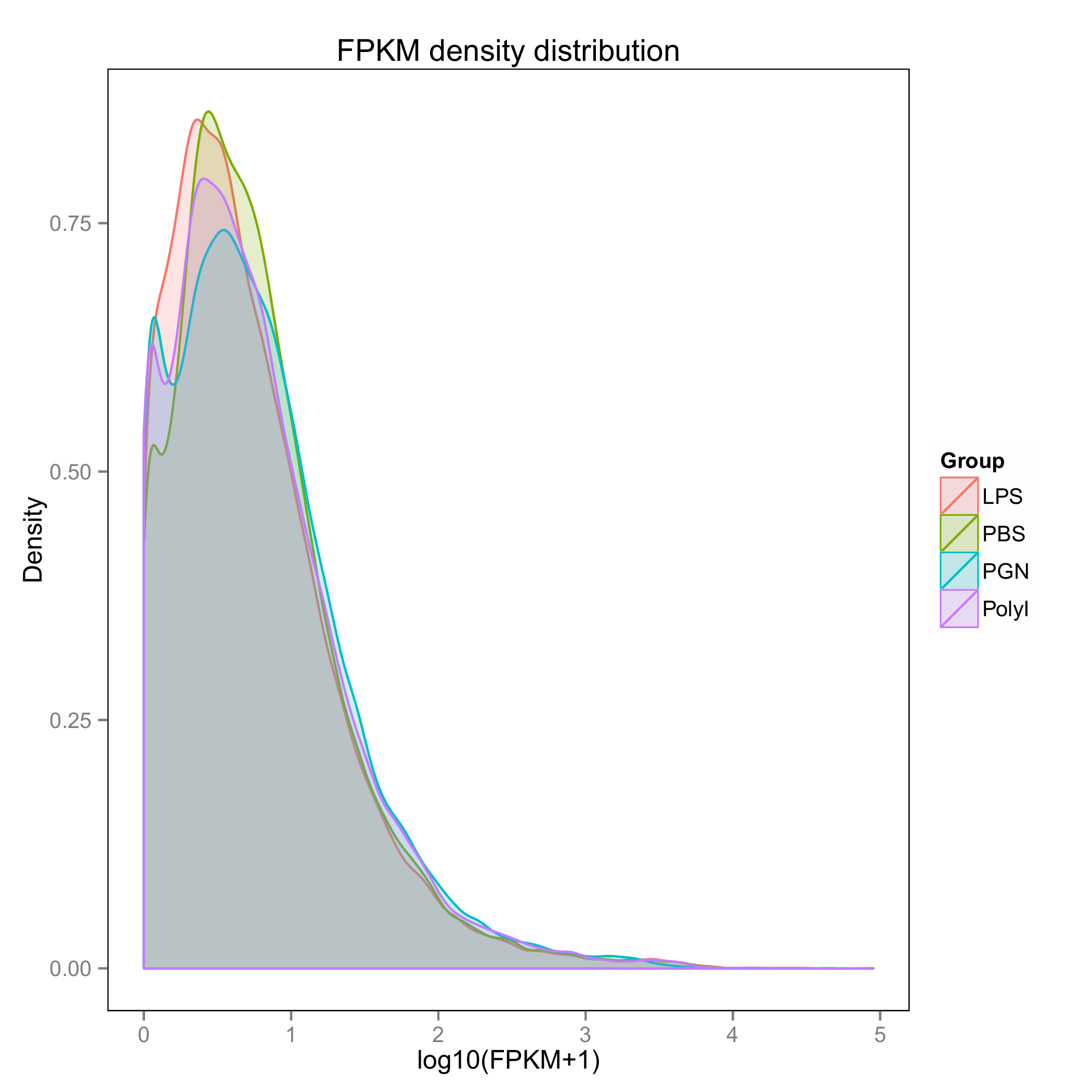

Supplement: Supplementary file 2 — Additional file 2. The density distribution of expression level of mapped clean read in LPS, PGN, poly(I:C), and PBS group. [file 12864_2020_6914_MOESM2_ESM.tif]
