# Supplementary material for: New insights into the Manila clam and PAMPs interaction based on RNA-seq analysis of clam through in vitro challenges with LPS, PGN, and poly(I:C)
Source: BMC Genomics. 2020 Aug 1;21:531. doi: 10.1186/s12864-020-06914-2 (PMC7430831; doi:10.1186/s12864-020-06914-2)
Supplement: Supplementary file 3 — Additional file 3. Kyoto Encyclopedia of Genes and Genomes (KEGG) assignment of unigenes in the transcriptome of R. philippinarum after LPS, PGN, poly(I:C) injection. [file 12864_2020_6914_MOESM3_ESM.docx]

Additional file 3: Kyoto Encyclopedia of Genes and Genomes (KEGG) assignment of unigenes in the transcriptome of *R. philippinarum* after LPS, PGN, poly(I:C) injection.

| **Pathway ID** | **Pathway** | ***P* value** |
| --- | --- | --- |
| LPS |  |  |
| map04111 | Cell cycle - yeast | 0.001718849 |
| map00624 | Polycyclic aromatic hydrocarbon degradation | 0.003527469 |
| map03430 | Mismatch repair | 0.004084647 |
| map04142 | Lysosome | 0.004847602 |
| map03420 | Nucleotide excision repair | 0.011149024 |
| map04810 | Regulation of actin cytoskeleton | 0.012697375 |
| map00363 | Bisphenol degradation | 0.021852373 |
| map00670 | One carbon pool by folate | 0.028502167 |
| map00627 | Aminobenzoate degradation | 0.035054574 |
| map02020 | Two-component system | 0.0366729 |
| map04964 | Proximal tubule bicarbonate reclamation | 0.036712593 |
| map04721 | Synaptic vesicle cycle | 0.038069866 |
| PGN |  |  |
| map04744 | Phototransduction | 3.09E-05 |
| map04740 | Olfactory transduction | 0.000400461 |
| map04114 | Oocyte meiosis | 0.000731204 |
| map05031 | Amphetamine addiction | 0.002470201 |
| map04621 | NOD-like receptor signaling pathway | 0.003077532 |
| map04626 | Plant-pathogen interaction | 0.003394983 |
| map04745 | Phototransduction - fly | 0.003988099 |
| map04910 | Insulin signaling pathway | 0.004929917 |
| map05034 | Alcoholism | 0.007491954 |
| map04728 | Dopaminergic synapse | 0.009953787 |
| map05214 | Glioma | 0.013377304 |
| map05164 | Influenza A | 0.019011055 |
| map04750 | Inflammatory mediator regulation of TRP channels | 0.019770903 |
| map01040 | Biosynthesis of unsaturated fatty acids | 0.02046122 |
| map04916 | Melanogenesis | 0.022298562 |
| map01230 | Biosynthesis of amino acids | 0.033793506 |
| map05206 | MicroRNAs in cancer | 0.034592929 |
| map04261 | Adrenergic signaling in cardiomyocytes | 0.04169302 |
| map05134 | Legionellosis | 0.044933626 |
| map04144 | Endocytosis | 0.04669602 |
| poly(I:C) |  |  |
| map00950 | Isoquinoline alkaloid biosynthesis | 0.000308372 |
| map05150 | Staphylococcus aureus infection | 0.001182821 |
| map00400 | Phenylalanine, tyrosine and tryptophan biosynthesis | 0.002273072 |
| map04918 | Thyroid hormone synthesis | 0.003115572 |
| map04514 | Cell adhesion molecules (CAMs) | 0.004507282 |
| map05020 | Prion diseases | 0.005176563 |
| map04662 | B cell receptor signaling pathway | 0.013951832 |
| map00960 | Tropane, piperidine and pyridine alkaloid biosynthesis | 0.01754872 |
| map03450 | Non-homologous end-joining | 0.023040046 |
| map00965 | Betalain biosynthesis | 0.035391938 |
| map04940 | Type I diabetes mellitus | 0.03657635 |
| map04340 | Hedgehog signaling pathway | 0.048975477 |
