# Supplementary material for: New insights into the Manila clam and PAMPs interaction based on RNA-seq analysis of clam through in vitro challenges with LPS, PGN, and poly(I:C)
Source: BMC Genomics. 2020 Aug 1;21:531. doi: 10.1186/s12864-020-06914-2 (PMC7430831; doi:10.1186/s12864-020-06914-2)
Supplement: Supplementary file 4 — Additional file 4. Primers used for qPCR in this study. [file 12864_2020_6914_MOESM4_ESM.docx]

Additional file 4. Primers used for qPCR in this study.

| **ID** | **Gene** | **Sequence** | |
| --- | --- | --- | --- |
| evm.model.xfSc0002255.2 | *RNF213* | Forward primer | CATTGCGGTTTGTTCCAAGTATCCTTC |
|  |  | Reverse primer | ATTCCTGATGCGTCTGACTGGTAAATC |
| evm.model.xfSc0000048.21 | *HSP70* | Forward primer | ATATGTCCTCCACTAGGTGATCCAGTC |
|  |  | Reverse primer | CCAAATGTCTTAGCCCAGTCATCTCC |
| Novel02352 | *GBP1* | Forward primer | GATACAGAGGGGATAGATGACCCAGAC |
|  |  | Reverse primer | TTAGACAAAAGCGTGGCAAGTATGAAC |
| evm.model.xfSc0002618.1 | *CALM* | Forward primer | GTACCAGCGGAGGGCGGATC |
|  |  | Reverse primer | TTCGTCATCACATAGCGTAACTCATCC |
| evm.model.xfSc0003071.3 | *IFI44* | Forward primer | AACGAACATGAACGATGGCTACCC |
|  |  | Reverse primer | GGCTCAACTGGCTCTTTTGTCTTTG |
| evm.model.xfSc0002514.1 | *Ubr2* | Forward primer | TTGTAGGCTTATCTAACTGGTCGCTTG |
|  |  | Reverse primer | GCTCTTCTTCATCCATATCCACTCGTG |
| Novel02605 | *ITGA* | Forward primer | TGGTTCCGTGTTCTACATGGCATTC |
|  |  | Reverse primer | CTCGCCGTCATCCATTGGGTTAG |
| evm.model.xfSc0001389.2 | *CNR3* | Forward primer | GGTGACGCAACCTGACAGAGAAG |
|  |  | Reverse primer | CAACCAAACAACGAGCTTGACCAC |
| evm.model.xfSc0000125.38 | *CTRP3* | Forward primer | TAATCATCGGTCTGGCAACTTCAGC |
|  |  | Reverse primer | TTCCCTCTTGTATTCTCTTTCCGTAGC |
| Novel04750 | *GBP1* | Forward primer | AAGGAGAAAGAAGACGAGCAAAGGC |
|  |  | Reverse primer | CTGTTGGTCTAGAGCTTCCGTTAGTG |
| evm.model.Sc0000062.24 | *HCK* | Forward primer | ACTTATTCCGATGTGTGGTCCTTTGG |
|  |  | Reverse primer | CTGGCATTTCCATCCTATACCCATCTG |
| Novel05234 | *HAAF* | Forward primer | CCACCACAATCACTATGAAGACAGGAG |
|  |  | Reverse primer | AGGCGTTGGTCCAGGGAGTATG |
| Novel07244 | *Ubr1* | Forward primer | CAGGTGCTGGATAACAGGGATTACTC |
|  |  | Reverse primer | ATCAGACACAGATTGACTGCTGGTATC |
| evm.model.xfSc0000743.13 | *CL12* | Forward primer | TGGGTGTCTCACAATACAAGATGCTAC |
|  |  | Reverse primer | TTCCTCTGCGGTTTCTATTTCTGCTAG |
| Novel04420 | *DD2* | Forward primer | ACACTTGCATTTGCGATGATGGATG |
|  |  | Reverse primer | TTACTAACAGCGTGGCGGAAGAAG |
| evm.model.xfSc0000599.7 | *LSS* | Forward primer | AATCGGCAGAACAGTCATCGGAAG |
|  |  | Reverse primer | TGTCGAGTCTGGCTTTGCTGATTC |
| Novel05650 | *DEF* | Forward primer | TGTGTTTCTCATAGTCGGTTACGCTTC |
|  |  | Reverse primer | TCAACAAGTAGCAGTGAGCAGAACAG |
| evm.model.xfSc0030245.1 | *LC* | Forward primer | ACATATTGGAGGACAGCGGAAAGAC |
|  |  | Reverse primer | TCATTTGGTGCCCATCTGAAGAAGG |
| evm.model.xfSc0000070.25 | *IFI* | Forward primer | AGCCAAATTTTCGAGCCAAATCTGC |
|  |  | Reverse primer | AGCGATGCCAGCGGAAGTAAATC |
| Novel06415 | *Siglec* | Forward primer | AAGTCTTCCTATAGTGCCAGCAAGTTG |
|  |  | Reverse primer | AGACATGAAGTAGTACACCAGCAAAGG |
| AY889707.1 | *β-actin* | Forward primer | CTCCCTTGAGAAGAGCTACGA |
|  |  | Reverse primer | GATACCAGCAGATTCCATACCC |
